# Supplementary material for: The Association of Folic Acid, Iron Nutrition during Pregnancy and Congenital Heart Disease in Northwestern China: A Matched Case-Control Study
Source: Nutrients. 2022 Oct 28;14(21):4541. doi: 10.3390/nu14214541 (PMC9657607; doi:10.3390/nu14214541)
Supplement: Supplementary file 1 [file nutrients-14-04541-s001.zip › nutrients-1934992-supplementary.pdf]

The Association of Folic Acid, Iron Nutrition during Pregnancy and Congenital Heart Disease in Northwestern China: A Matched Case-Control Study

Ming-Xin Yan [1], Yan Zhao [2], Dou-Dou Zhao [3], Shao-Nong Dang [1], Ruo Zhang [1], Xin-Yu Duan [1], Pei-Xi Rong [1], Yu-Song Dang [1], Lei-Lei Pei [1,*] and Peng-Fei Qu [2,*]

[1] Department of Epidemiology and Biostatistics, School of Public Health, Xi'an Jiaotong University Health Science Center, Xi'an 710061, China

[2] The First Affiliated Hospital of Xi'an Jiaotong University Dermatology, Xi'an 710061

[3] ChinaTranslational Medicine Center Northwest Women's and Children's Hospital, Xi'an 710061, China

*Corresponding: pll_paper@126.com (L.-L.P.); xinxi3057@163.com (P.-F.Q.)

Supplementary Table S1. Types of congenital heart disease.

Congenital heart disease	Number	Proportion (%)
Ventricular septal defect (VSD)	187	31.17
Atrial septal defect (ASD)	75	12.50
Patent ductus arteriosus (PDA)	75	12.50
Atrioventricular septal defect (AVSD)	51	8.50
Tetralogy of Fallot (TOF)	45	7.50
Congenital aortic (valve) stenosis	14	2.33
Pulmonary artery (valve) stenosis	39	6.50
Aortic constriction	10	1.67
Transposition of the aorta	9	1.50
Pulmonary artery atresia	5	0.83
Tricuspid atresia	14	2.33
Arterial Stem Forever	3	0.50
Hypoplastic left heart syndrome	12	2.00
Interrupted aortic arch	9	1.50
Others	52	8.67
Total	600	100.00

Supplementary Table S2. Interaction of maternal folic acid and iron nutrition in incidence of different subtypes of CHD.

Variables	Folic acid supplementation [c]	VSD(N=187) [d] OR (95%CI)	ASD(N=75) [d] OR (95%CI)	PDA(N=75) [d] OR (95%CI)
Iron supplementation [a]				
0	0	1.00	1.00	1.00
1	0	0.30(0.08, 1.12)	0.21(0.04, 1.10)	0.12(0.02, 0.99)
0	1	0.54(0.25, 1.15)	0.47(0.19, 1.21)	0.34(0.13, 0.89)
1	1	0.21(0.09, 0.51)	0.15(0.04, 0.51)	0.23(0.08, 0.72)
Dietary iron intake [b]				
0	1	1.00	1.00	1.00
1	0	0.46(0.10, 2.11)	0.93(0.17, 5.00)	0.79(0.17, 3.66)
0	1	0.57(0.27, 1.19)	0.61(0.23, 1.61)	0.58(0.19, 1.72)
1	1	0.42(0.19, 0.97)	0.51(0.17, 1.59)	0.22(0.06, 0.81)

[a] 0 = No and 1 = Yes. [b] 0= ”<29 mg/d”,1= ”≥29 mg/d”. [c] 0 = No and 1 = Yes. [d] Adjusted for age, ethnicity, education, residence, wealth index, cold in early pregnancy, fever in early pregnancy, drinking, passive smoke, hair dyeing and perming, gravidity.

Supplementary Table S3. Multiplication and additive interaction of folic acid and iron nutrition in incidence of different subtypes of CHD.

Variables	Interactions	VSD(N=187) [d]	ASD(N=75) [d]	PDA(N=75) [d]
Multiplication model	Folic acid supplementation ×Iron supplementation	0.36(0.20, 0.65)	0.30(0.12, 0.76)	0.60(0.27, 1.32)
Additive model	RERI[a]	0.37(-0.08, 0.82)	0.46(-0.13, 1.05)	0.77(0.40, 1.14)
	S[b]	0.68(0.48, 0.96)	0.65(0.43, 0.99)	0.50(0.38, 0.65)
	AP[c]	1.78(-1.60, 5.16)	3.19(-3.58, 9.96)	3.30(-1.02, 7.61)
Multiplication model	Folic acid supplementation ×Dietary iron intake	0.71(0.43, 1.17)	0.72(0.33, 1.59)	0.33(0.13, 0.81)
Additive model	RERI	0.40(-0.38, 1.18)	-0.02 (-1.64, 1.59)	-0.14(-1.64, 1.35)
	S	0.29(0.28, 1.26)	1.05(0.03, 35.34)	1.22(0.11, 13.36)
	AP	0.94(-1.28, 3.15)	-0.05 (-3.18, 3.08)	-0.65(-7.01, 5.71)

[a] The relative excess risk due to interaction. [b] The attributable proportion. [c] The synergy index.

[d] Adjusted for age, ethnicity, education, residence, wealth index, cold in early pregnancy, fever in early pregnancy, drinking, passive smoke, hair dyeing and perming, gravidity.

Supplementary Table S4. Interaction of folic acid and iron supplements in incidence of CHD by different sociodemographic characteristics.

Variables	Folic acid supplementation [a]	Iron supplementation [b]	N n (%)	Cases n (%)	Controls n (%)	Model[c] OR (95%CI)
Age						
<30	0	0	140(11.82)	73(19.31)	67(8.31)	1.00
	0	1	50(4.22)	13(3.44)	37(4.59)	0.18(0.07, 0.48)
	1	0	693(58.53)	252(66.67)	441(54.71)	0.52(0.32, 0.85)
	1	1	301(25.42)	40(10.58)	261(32.38)	0.16(0.09, 0.29)
≥30	0	0	88(14.29)	47(21.17)	41(10.41)	1.00
	0	1	27(4.38)	8(3.60)	19(4.82)	0.71(0.16, 3.11)
	1	0	349(56.65)	142(63.96)	207(52.54)	1.06(0.47, 2.37)
	1	1	152(24.68)	25(11.27)	127(32.23)	0.33(0.12, 0.90)
Education						
Senior high school or lower	0	0	106(19.85)	74(28.03)	32(11.85)	1.00
	0	1	24(4.49)	9(3.41)	15(5.56)	0.10(0.01, 1.21)
	1	0	316(59.18)	169(64.02)	147(54.44)	0.66(0.24, 1.82)
	1	1	88(16.48)	12(4.55)	76(28.15)	0.07(0.02, 0.32)
College or above	0	0	122(9.64)	46(13.69)	76(8.17)	1.00
	0	1	53(4.19)	12(3.57)	41(4.41)	0.34(0.14, 0.87)
	1	0	726(57.35)	225(66.96)	501(53.87)	0.56(0.33, 0.93)

	1	1	365(28.83)	53(15.77)	312(33.55)	0.23(0.13, 0.42)
Residence						
Urban	0	0	98(9.79)	30(14.93)	68(8.50)	1.00
	0	1	38(3.80)	6(2.99)	32(4.00)	0.35(0.10, 1.16)
	1	0	566(56.54)	129(64.18)	437(54.63)	0.70(0.36, 1.35)
	1	1	299(29.87)	36(17.91)	263(32.87)	0.35(0.16, 0.76)
Rural	0	0	130(16.27)	90(22.56)	40(10.00)	1.00
	0	1	39(4.88)	15(3.76)	24(6.00)	0.25(0.08, 0.86)
	1	0	476(59.57)	265(66.42)	211(52.75)	0.68(0.38, 1.20)
	1	1	154(19.27)	29(7.27)	125(31.25)	0.18(0.08, 0.39)
Wealth index						
Poor	0	0	106(16.46)	74(23.64)	32(9.67)	1.00
	0	1	34(5.28)	14(4.47)	20(6.04)	0.11(0.02, 0.56)
	1	0	396(61.49)	207(66.13)	189(57.10)	0.34(0.13, 0.91)
	1	1	108(16.77)	18(5.75)	90(27.19)	0.07(0.02, 0.26)
Moderate	0	0	58(10.27)	27(16.17)	31(7.79)	1.00
	0	1	15(2.65)	5(2.99)	10(2.51)	0.47(0.04, 6.06)
	1	0	320(56.64)	108(64.67)	212(53.27)	0.53(0.17, 1.66)
	1	1	172(30.44)	27(16.17)	145(36.43)	0.30(0.09, 0.99)
Rich	0	0	64(10.83)	19(15.83)	45(9.55)	1.00
	0	1	28(7.74)	2(1.67)	26(5.52)	0.22(0.02, 2.25)
	1	0	326(55.16)	79(65.83)	247(52.44)	0.86(0.32, 2.33)

	1	1	173(29.27)	20(16.67)	153(32.48)	0.31(0.09, 1.00)

[a] 0 = No and 1 = Yes. [b] 0 = No and 1 = Yes. [c] Adjusted for covariates other than subgroup variables.

Supplementary Table S5. Multiplicative and additive interaction indicators of folic acid and iron supplements in incidence of CHD by different sociodemographic characteristics.

Variables		Interactions	Model[d]
Age			
<30	Multiplication model	Folic acid supplementation × Iron supplementation	0.28(0.18, 0.45)
	Additive model	RERI[a]	0.45(0.14, 0.77)
		S[b]	0.65(0.52, 0.82)
		AP[c]	2.84(-0.27, 5.96)
≥30	Multiplication model	Folic acid supplementation × Iron supplementation	0.30(0.15, 0.62)
	Additive model	RERI	-0.44(-1.84, 0.97)
		S	-[e]
		AP	-1.31(-5.20, 2.59)
Education			
Senior high school or lower	Multiplication model	Folic acid supplementation × Iron supplementation	0.10(0.03, 0.37)
	Additive model	RERI	0.31(-0.44, 1.06)
		S	0.75(0.42, 1.35)

		AP	4.69(-11.20, 20.59)
College or above	Multiplication model	Folic acid supplementation×Iron supplementation	0.39(0.26, 0.59)
	Additive model	RERI	0.33(-0.11, 0.77)
		S	0.70(0.48, 1.02)
		AP	1.42(-0.95, 3.79)
Residence			
Urban	Multiplication model	Folic acid supplementation×Iron supplementation	0.47(0.29, 0.78)
	Additive model	RERI	0.31(-0.29, 0.90)
		S	0.68(0.38, 1.20)
		AP	0.88(-1.21, 2.97)
Rural	Multiplication model	Folic acid supplementation×Iron supplementation	0.28(0.15, 0.50)
	Additive model	RERI	0.25(-0.27, 0.76)
		S	0.77(0.48, 1.23)
		AP	1.38(-2.10, 4.86)
Wealth index			
Poor	Multiplication model	Folic acid supplementation×Iron supplementation	0.20(0.08, 0.51)
	Additive model	RERI	0.61(0.21, 1.02)
		S	0.60(0.47, 0.77)

		AP	8.97(-7.10, 25.04)
Moderate	Multiplication model	Folic acid supplementation×Iron supplementation	0.50(0.24, 1.05)
	Additive model	RERI	0.29(-1.04, 1.62)
		S	0.71(0.19, 2.57)
		AP	0.98(-4.04, 6.01)
Rich	Multiplication model	Folic acid supplementation×Iron supplementation	0.36(0.16, 0.82)
	Additive model	RERI	0.23(-0.72, 1.17)
		S	0.75(0.28, 2.00)
		AP	0.74(-2.80, 4.29)

[a] The relative excess risk due to interaction. [b] The attributable proportion. [c] The synergy index. [d] Adjusted for covariates other than subgroup variables. [e]. Missing data.

Supplementary Table S6. Interaction of maternal folic acid supplement and dietary iron intake in incidence of CHD by different sociodemographic characteristics.

Variables	Folic acid supplementation [a]	Dietary iron intake [b]	N n (%)	Cases n (%)	Controls n (%)	Model[c] OR (95%CI)
Age						
<30	0	0	127(10.73)	66(17.46)	61(7.57)	1.00
	0	1	63(5.32)	20(5.29)	43(5.33)	0.28(0.12, 0.68)
	1	0	629(53.12)	202(53.44)	427(52.98)	0.42(0.25, 0.70)

	1	1	365(30.83)	90(23.81)	275(34.12)	0.35(0.19, 0.63)
≥30	0	0	82(13.31)	43(19.37)	39(9.90)	1.00
	0	1	33(5.36)	12(5.41)	21(5.33)	0.97(0.23, 4.13)
	1	0	317(51.46)	123(55.40)	194(49.24)	0.99(0.40, 2.42)
	1	1	184(29.87)	44(19.82)	140(35.53)	0.59(0.21, 1.68)
Education						
Senior high school or lower	0	0	96(17.98)	67(25.38)	29(10.74)	1.00
	0	1	34(6.37)	16(6.06)	18(6.67)	0.29(0.05, 1.80)
	1	0	274(51.31)	132(50.00)	142(52.59)	0.90(0.31, 2.59)
	1	1	130(24.34)	49(18.56)	81(30.00)	0.31(0.11, 0.89)
College or above	0	0	113(8.93)	42(12.50)	71(7.63)	1.00
	0	1	62(4.90)	16(4.76)	46(4.95)	0.41(0.17, 0.98)
	1	0	672(53.08)	193(57.44)	479(51.51)	0.48(0.27, 0.85)
	1	1	419(33.09)	85(25.30)	334(35.91)	0.29(0.15, 0.54)
Residence						
Urban	0	0	81(8.09)	25(12.44)	56(7.00)	1.00
	0	1	55(5.49)	11(5.47)	44(5.50)	0.45(0.15, 1.35)
	1	0	521(52.05)	116(57.71)	405(50.63)	0.74(0.34, 1.58)
	1	1	344(34.37)	49(24.38)	295(36.87)	0.42(0.19, 0.93)
Rural	0	0	128(16.02)	84(21.05)	44(11.00)	1.00
	0	1	41(5.13)	21(5.26)	20(5.00)	0.95(0.33, 2.70)

	1	0	425(53.19)	209(52.38)	216(54.00)	0.73(0.41, 1.32)
	1	1	205(25.66)	85(21.30)	120(30.00)	0.55(0.28, 1.09)
Wealth index						
Poor	0	0	116(18.01)	75(23.96)	41(12.39)	1.00
	0	1	24(3.73)	13(4.15)	11(3.32)	0.78(0.12, 4.98)
	1	0	338(52.48)	159(50.80)	179(54.08)	0.56(0.25, 1.26)
	1	1	166(25.78)	66(21.09)	100(30.21)	0.43(0.17, 1.08)
Moderate	0	0	41(7.26)	20(11.98)	21(5.27)	1.00
	0	1	32(5.66)	12(7.19)	20(5.03)	0.32(0.05, 2.00)
	1	0	297(52.57)	98(58.68)	199(50.00)	0.53(0.14, 1.95)
	1	1	195(34.51)	37(22.15)	158(39.70)	0.16(0.04, 0.64)
Rich	0	0	52(8.80)	14(11.67)	38(8.07)	1.00
	0	1	40(6.77)	7(5.83)	33(7.01)	0.57(0.12, 2.74)
	1	0	311(52.62)	68(56.67)	243(51.59)	0.78(0.27, 2.28)
	1	1	188(31.81)	31(25.83)	157(33.33)	0.65(0.19, 2.25)

[a] 0 = No and 1 = Yes. [b] 0= ”<29 mg/d”,1= ”≥29 mg/d”. [c] Adjusted for covariates other than subgroup variables.

Supplementary Table S7. Multiplication and additive interaction indicators of folic acid supplement and dietary iron intake on CHD by different sociodemographic characteristics.

Variables	Interactions	Model[d]
Age		

<30	Multiplication model	Folic acid supplementation×Dietary iron intake	0.79(0.60, 1.00)
	Additive model	RERI[a]	0.65(0.35, 0.94)
		S[b]	0.50(0.40, 0.63)
		AP[c]	1.86(0.28, 3.44)
≥30	Multiplication model	Folic acid supplementation×Dietary iron intake	0.63(0.46, 0.88)
	Additive model	RERI	-0.37(-2.08, 1.35)
		S	-[e]
		AP	-0.62(-3.38, 2.14)
Education			
Senior high school or lower	Multiplication model	Folic acid supplementation×Dietary iron intake	0.71(0.52, 0.97)
	Additive model	RERI	0.12(-0.96, 1.18)
		S	0.86(0.24, 3.12)
		AP	0.38(-3.33, 4.08)
College or above	Multiplication model	Folic acid supplementation×Dietary iron intake	0.74(0.57, 0.94)
	Additive model	RERI	0.40(-0.04, 0.84)
		S	0.64(0.45, 0.92)
		AP	1.38(-0.74, 3.51)
Residence			
Urban	Multiplication model	Folic acid supplementation×Dietary iron intake	0.62(0.45, 0.86)
	Additive model	RERI	0.23(-0.47, 0.94)
		S	0.71(0.33, 1.54)

		AP	0.56(-1.45, 2.58)
Rural	Multiplication model	Folic acid supplementation×Dietary iron intake	0.81(0.64, 1.03)
	Additive model	RERI	-0.13(-1.21, 0.96)
		S	1.40(0.04, 46.22)
		AP	-0.23(-2.14, 1.68)
Wealth index			
Poor	Multiplication model	Folic acid supplementation×Dietary iron intake	0.84(0.64, 1.11)
	Additive model	RERI	0.09(-1.44, 1.62)
		S	0.86(0.09, 8.39)
		AP	0.22(-3.43, 3.86)
Moderate	Multiplication model	Folic acid supplementation×Dietary iron intake	0.55(0.38, 0.80)
	Additive model	RERI	0.31(-0.64, 1.25)
		S	0.73(0.34, 1.59)
		AP	1.96(-6.20, 10.12)
Rich	Multiplication model	Folic acid supplementation×Dietary iron intake	0.77(0.51, 1.16)
	Additive model	RERI	0.30(-0.77, 1.36)
		S	0.54(0.10, 2.77)
		AP	0.46(-1.44, 2.36)

[a] The relative excess risk due to interaction. [b] The attributable proportion. [c] The synergy index. [d] Adjusted for covariates other than subgroup variables. [e] Missing data.
